# Investigation of the morphological, physiological, biochemical, and catabolic characteristics and gene expression under drought stress in tolerant and sensitive genotypes of wild barley [*Hordeum vulgare subsp. spontaneum* (K. Koch) Asch. & Graebn.]

**DOI:** 10.1186/s12870-024-04894-z

**Published:** 2024-03-26

**Authors:** Hooman Shirvani, Ali Ashraf Mehrabi, Mohsen Farshadfar, Hooshmand Safari, Ali Arminian, Foad Fatehi, Alireza Pouraboughadareh, Peter Poczai

**Affiliations:** 1 Department of Agronomy and Plant Breeding, Faculty of Agriculture, Ilam University, Ilam, Iran; 2 Research Center of Medicinal Plants, Shahed University, Tehran, Iran; 3 Forests and Rangelands Research Department, Agricultural Research and Training Center and Kermanshah Province, Agricultural Research, Education and Extension Organization, Kermanshah, Iran; 4 Department of Agriculture, Payame Noor University, Tehran, Iran; 5 Seed and Plant Improvement Institute, Agricultural Research, Education and Extension Organization (AREEO), Karaj, Iran; 6 Botany and Mycology Unit, Finnish Museum of Natural History, University of Helsinki, Helsinki, Finland

**Keywords:** *Hordeum vulgare*, Drought stress, Stomatal analysis, Gene expression

## Abstract

**Background:**

Barley (*H. vulgare L*.) is an important cereal crop cultivated across various climates globally. Barley and its ancestor (*H. vulgare subsp. spontaneum*) are an economically valuable model for genetic research and improvement. Drought, among various abiotic stresses, is a substantial threat to agriculture due to its unpredictable nature and significant impact on crop yield.

**Results:**

This study was conducted in both greenhouse and laboratory settings. Prior to the study, wild barley accessions were pre-selected based on their sensitivity or tolerance to drought as determined from fieldwork in the 2020–2021 and 2021–2022 cropping seasons. The effects of three levels of drought stress were evaluated (control, 90–95% field capacity [FC]; mild stress, 50–55% FC; and severe stress, 25–30% FC). Several parameters were assessed, including seedling and root growth, enzymatic activity (CAT, SOD, POD), soluble protein levels, chlorophyll content, carotenoids, abaxial and adaxial stomatal density and dimensions, and relative gene expression of *Dhn1*, *SOD*, *POD*, and *CAT*. Drought stress significantly increased enzyme activities, especially at 25–30% FC, and more in the tolerant genotype. On the other hand, sensitive genotypes showed a notable increase in stomatal density. Under drought stress, there was a general decline in seedling and root growth, protein content, chlorophyll and carotenoids, and stomatal dimensions. Importantly, gene expression analysis revealed that *Dhn1*, *SOD*, *POD*, and *CAT* were upregulated under drought, with the highest expression levels observed in the drought-tolerant genotype under severe stress conditions (25–30% FC).

**Conclusions:**

Our investigation highlights the distinct morphological, physiological, biochemical, and gene-expression profiles of drought-resistant and drought-sensitive wild barley genotypes under varying degrees of drought.

**Supplementary Information:**

The online version contains supplementary material available at 10.1186/s12870-024-04894-z.

## Background

Barley (*H. vulgare* L.) and its ancestor agronomy (*H. vulgare subsp. spontaneum*) are excellent economic model systems for genetic exploration and exploitation. Both species are diploid and can be crossed with each other. A set of genomic tools, including linkage maps, QTL data, ESTs, BAC libraries, and arrays to analyze the H genome (homologous to the genomes of A, B, and D hexaploid wheat) are available [1]. The western regions of Iran, the Middle East, and the Fertile Crescent serve as the primary hubs for wild barley diversity [2, 3].

In recent years, climate change has had a significant impact on the production of agricultural products. This is primarily due to the emergence of abiotic stresses, such as drought, heat, and salinity. Among these stresses, drought or dehydration is particularly challenging as it is unpredictable in its occurrence, intensity, and duration [4]. Drought stress in barley leads to various changes in morphology, physiology, biochemistry, and catabolic processes. Barley plants respond to drought stress by modifying morphology, anatomy, and physiology to enhance water usage efficiency and limit water loss through transpiration [5]. Drought stress negatively affects chlorophyll content and photosynthetic efficiency, leading to a decline in plant growth and yield [6]. Drought tolerance in barley is a complex trait that involves multiple mechanisms, including escape, avoidance, and tolerance [7].

Understanding the molecular dynamics and genetic composition underlying drought tolerance is crucial for developing drought-resistant barley varieties [8]. Recent advances in genomics and transcriptomics have provided insights into the genetic and transcription factors involved in drought resistance in barley [9]. Evaluation and identification of wild relative species of barley, which are adapted to unfavorable environmental conditions in various geographical areas, is one of the fundamental steps in producing and breeding cultivars that are tolerant to drought. Given the role and importance of wild species, it is necessary to understand the distribution and amount of genetic diversity of these species for different traits so that they can be effectively and efficiently used in plant breeding [10].

Current breeding programs for drought tolerance are based on identifying morphological, physiological, and biochemical traits that are related to drought tolerance. The next step is to identify the specific genes involved in these traits and transfer them to agricultural cultivars [11]. Gene expression in response to drought stress in barley has been extensively studied. Several studies have identified differentially expressed genes in barley under drought stress [12]. Manh et al. [13]found that overexpression of the *WHIRLY1* gene in barley delayed the onset of senescence and suppressed expression of drought-related marker genes. Alamholo and Tarinejad [14] performed a meta-analysis of microarray data and identified numerous upregulated and downregulated genes related to drought tolerance in barley. Additionally, Wang et al. [6] used DNA affinity purification sequencing (DAP-seq) to identify novel transcription factors involved in drought resistance in highland barley.

Stomata closure and preventing water escape via evaporation from the leaf surface are primary plant responses to drought stress [15]. One of the most important biochemical changes in plants in response to drought stress is production of reactive oxygen species (ROS). These include superoxide radicals (O_2_^–^), hydroxyl radicals (OH^−^), hydrogen peroxide radicals (H_2_O_2_), and Alcozy radicals (RO^−^), and other non-radical agents like hydrogen peroxide and singlet oxygen [16]. When faced with stress, plants employ various mechanisms to scavenge reactive oxygen species and protect themselves from the detrimental effects caused by these species [17]. The levels of reactive oxygen species (ROS) in biological systems are regulated by enzymatic and non-enzymatic antioxidant defenses. Enzyme systems consist of superoxide dismutase (SOD), catalase (CAT), peroxidase (POD), and ascorbate peroxidase (APX) [18]. Antioxidant enzymes can directly and indirectly lead to increased stress tolerance.

Farooq et al. [19] also observed that increasing the level of antioxidants by scavenging reactive oxygen species leads to improved drought tolerance. In recent years, progress in breeding methods and genetic engineering, such as expression of biosynthesis genes for osmotic protection, oxygen scavenging system, molecular homogenization, translocation, and gene transfer have provided new approaches in the development and production of drought-tolerant cultivars. Therefore, proper implementation of molecular breeding and biotechnology programs requires an understanding of the tolerance mechanism(s) in agricultural plants and their wild relatives and the evaluation and identification of superior genotypes.

Tolerance to drought stress in plants is a relative state. By evaluating the activity level of antioxidant systems and examining other morphological, physiological, and catabolic traits under drought stress conditions, genotypes or plant materials can be identified as superior to other samples. Therefore, a better understanding of the morphological, physiological, biochemical, and catabolic responses and identification of genomic regions related to drought stress can assist breeders in programs aimed at improving drought tolerance or developing new varieties. To the best of our knowledge, no previous research has been conducted on the antioxidant, morphological, physiological, and catabolic properties of this wild barley germplasm (*H. vulgare subsp. spontaneum*).

## Results

### Selection of tolerant and sensitive genotypes to drought stress

Based on the stress-tolerance scoring (STS) index, genotype numbers 88, 86, 97, 62, 113, 12, 85, 74, and 73 are tolerant and genotype numbers 72, 25, 51, 103, 56, 1, 67, 26, 17, 13, and 18 are sensitive (Table 1). Based on the results of performance-based indicators (SSI, TOL, MP, GMP, STI, and HAM) in the field, genotypes tolerant (88-Kozran, Kermanshah, 34.4965° N, 46.5982° E, MSL: 1368, Accession code: IUGB-01657) and sensitive (72-Muchesh, Kurdistan: 35.0571° N, 47.1522° E, MSL: 1368, Accession code: IUGB-01975) to drought stress were selected using the STS index. A notable feature of the STS index is that it is not only used for determining a resistance index; but several other indices can also be considered.

**Table 1:** STS index and rank of wild barley genotypes in the field

Genotype	STS	Rank	Genotype	STS	Rank	Genotype	STS	Rank
1	-2.973	109	39	0.062	88	77	4.820	35
2	0.839	77	40	0.538	81	78	9.987	10
3	0.778	78	41	-1.936	103	79	4.872	34
4	2.643	59	42	4.104	41	80	2.194	66
5	3.850	45	43	3.001	52	81	2.445	61
6	-1.702	100	44	4.894	33	82	4.446	37
7	1.486	71	45	2.957	53	83	3.982	43
8	5.687	25	46	3.021	51	84	8.334	14
9	-1.557	99	47	4.041	42	85	10.931	7
10	3.933	44	48	2.673	58	86	17.793	2
11	-0.143	90	49	0.272	83	87	8.475	12
12	11.291	6	50	-1.762	101	88	17.819	1
13	-2.270	105	51	-4.678	112	89	4.779	36
14	0.484	82	52	5.381	28	90	0.110	87
15	8.137	15	53	2.237	65	91	0.547	80
16	7.456	17	54	5.375	29	92	3.642	47
17	-2.608	106	55	-1.200	96	93	1.396	72
18	-2.012	104	56	-4.499	110	94	2.762	56
19	-0.225	91	57	6.011	23	95	1.716	68
20	5.173	30	58	1.382	73	96	-0.854	93
21	2.369	62	59	4.405	38	97	17.434	3
22	7.220	19	60	-1.354	97	98	1.570	70
23	0.879	76	61	8.948	11	99	1.613	69
24	1.025	75	62	15.207	4	100	1.252	74
25	-4.912	113	63	3.360	50	101	5.573	26
26	-2.823	107	64	2.868	55	102	-0.868	94
27	2.512	60	65	6.106	21	103	-4.675	111
28	-0.792	92	66	7.448	18	104	-0.101	89
29	-1.924	102	67	-2.954	108	105	1.850	67
30	6.103	22	68	-0.909	95	106	0.209	85
31	4.397	39	69	3.404	49	107	2.274	63
32	7.746	16	70	4.974	32	108	4.995	31
33	6.233	20	71	0.268	84	109	2.259	64
34	4.341	40	72	-6.041	114	110	5.798	24
35	8.337	13	73	10.174	9	111	3.571	48
36	2.759	57	74	10.359	8	112	0.577	79
37	-1.370	98	75	2.939	54	113	13.467	5
38	3.753	46	76	5.498	27	114	0.141	86

### Gene expression

The expression of *Dhn1*, *SOD*, *POD*, and *CAT* genes was investigated. The replication curves of the studied genes, as well as a reference gene (*α-tubulin*), showed successful and appropriate replication. Additionally, the replication process exhibited the absence of non-specific replication with increasing replication cycles.

The results of the variance analysis for the relative expression data are shown in Table 2. Notably, significant differences were observed in the levels of drought stress, different genotypes, and the interaction between drought stress and genotype for all genes.

**Table 2:** Variance analysis of relative gene expression of tolerant and sensitive genotypes at different levels of drought stress

Sources of variation	Degrees of freedom	Mean Square			
		Dhn1	SOD	POD	CAT
Genotype (G)	1	22.728**	18.952**	3.454**	27.877**
Stress treatment (S)	2	17.42**	16.628**	6.327**	12.034**
G × S	2	9.017**	7.635**	0.707**	6.477**
Error	18	0.041	0.043	0.018	0.036
CV%		11.89%	12.10%	9.45%	11.54%

* and ** indicate significance at the 5% and 1% probability levels, respectively.

**Fig. 1:**
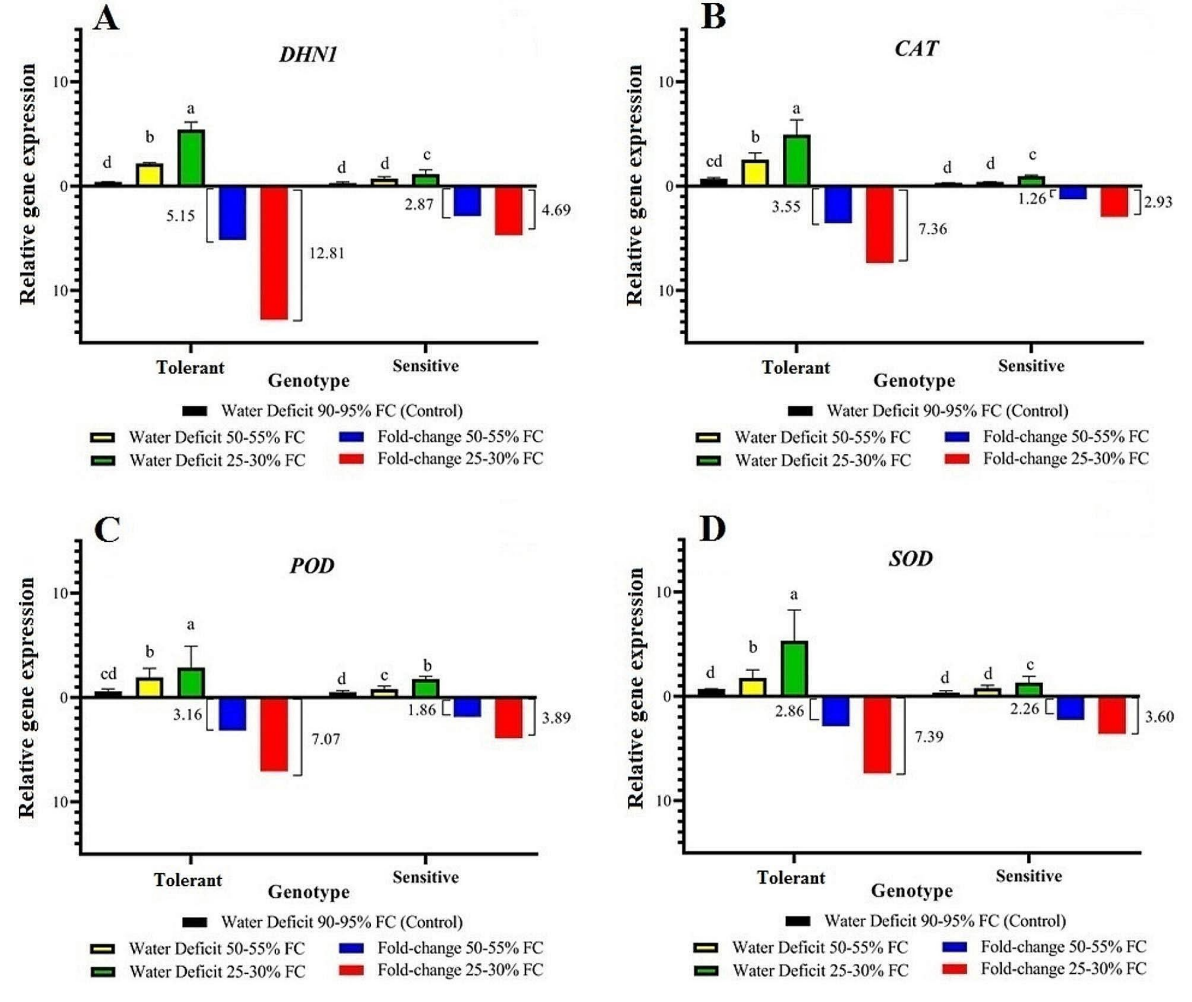
Relative gene-expression levels in sensitive and tolerant genotypes under drought stress and control conditions

Mean comparison revealed that the relative expression of the *Dhn1*, *SOD*, *POD*, and *CAT* genes increased under drought stress compared with control conditions. The tolerant-genotype showed the highest expression at the 25–30% FC conditions compared to the control conditions. Specifically, drought stress conditions (25–30% and 50–55% FC) increased expression of the *Dhn1* gene by 12.51- and 5.15-fold compared to the control conditions, respectively. In contrast, the susceptible genotype showed a minimum change (2.86-fold at 25–30% FC and a 4.69-fold at 50–55% FC) than the control conditions (Fig. 1-A).

The drought-tolerant genotype showed the highest relative expression of *CAT* compared with the sensitive genotype. In the tolerant genotype when compared with control conditions, the most significant changes in *CAT* expression under drought conditions occurred at 25–30% FC (7.36-fold) followed by 50–55% FC (3.55-fold) (Fig. 1-B).

The tolerant genotype exhibited the highest level of *POD* expression under stress conditions compared with the sensitive genotype. The most significant changes in *POD* expression when compared with control conditions occurred at a stress level 25–30% FC (7.07-fold) in the tolerant genotype and the sensitive genotype (3.89-fold) (Fig. 1-C).

The trend of changes in *SOD* gene expression varied. At a stress level of 25–30% FC, the tolerant genotype exhibited the highest level of gene expression compared to the non-drought stress conditions. Additionally, the sensitive genotype showed the highest increase in gene expression after tolerant genotype. Specifically, the sensitive genotype had a greater increase in gene expression at the stress level of 25–30% FC compared to the non-drought stress conditions (tolerant 7.39-fold and sensitive 3.60-fold) (Fig. 1-D).

### Morphological traits

The results of ANOVA revealed a significant difference between stress levels, genotype evaluations, and the interaction between drought stress and genotypes in root length, fresh weight, dry weight, seedling length, fresh weight, and dry weight. These differences were significant at the 5% level for root length, seedling length, and seedling fresh weight and at the 1% level for the other traits (Table 3).

**Table 3:** Variance analysis of morphological and root traits for sensitive and tolerant genotypes at different levels of drought stress

Sources of variation	Degrees of freedom	Mean Square					
		Root length	Root fresh weight	Root dry weight	Seedling length	Seedling fresh weight	Seedling dry weight
Genotype (G)	1	117.556*	0.111**	0.002**	40.5**	0.333**	0.804**
Stress treatment (S)	2	627.056**	0.289**	0.021**	312**	2.379**	1.502**
G × S	2	40.056*	0.008**	0.001**	6*	0.023*	0.05**
Error	12	8.889	0.001	0.000001	1.5	0.005	0.003
CV%		4.92%	6.50%	5.57%	3.55%	4.91%	5.35%

* and ** indicate significance at the 5% and 1% probability levels, respectively.

**Fig. 2:**
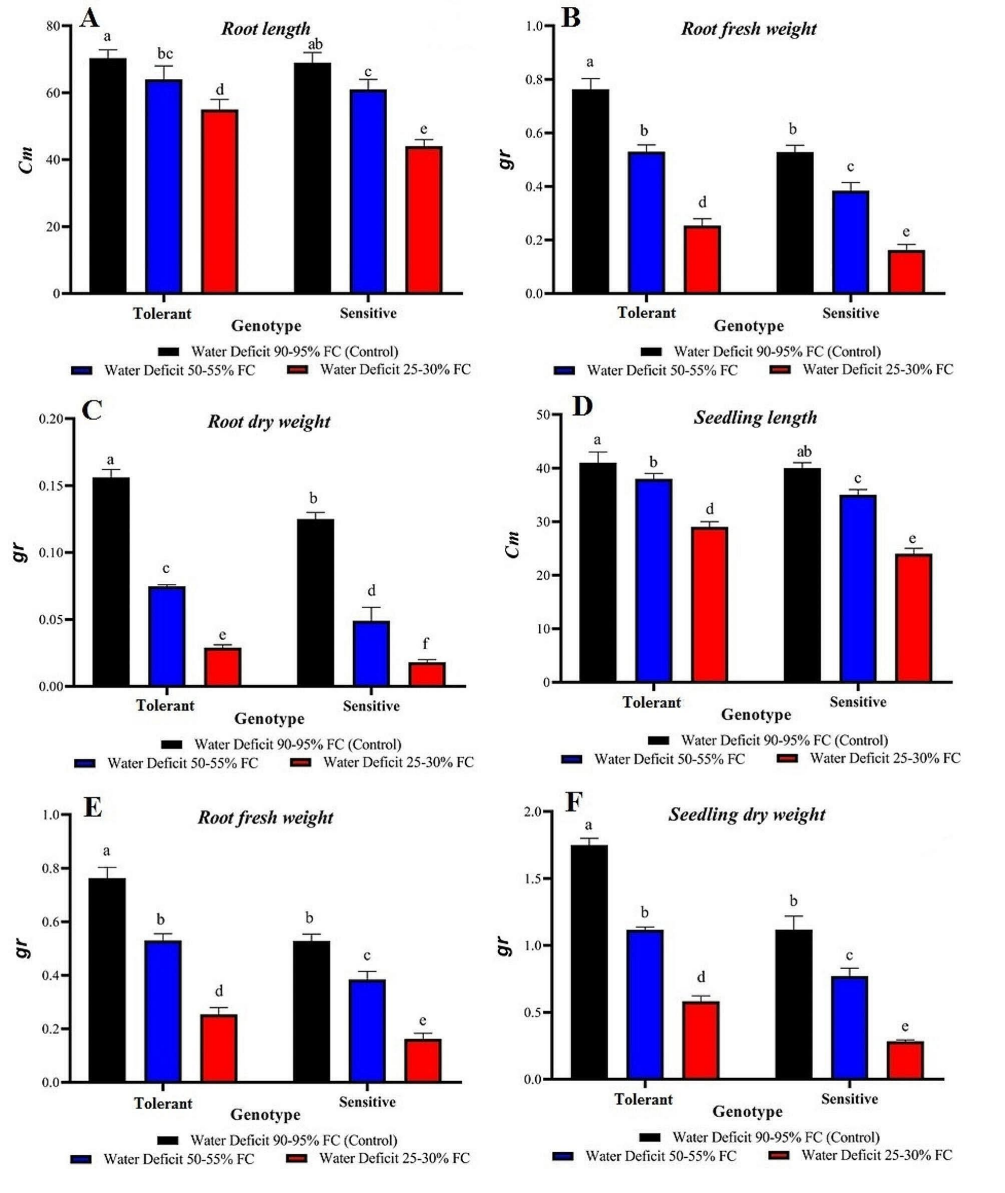
comparison of seedling and root traits in sensitive and tolerant genotypes under drought stress and control conditions

The tolerant genotype exhibited greater resistance to drought stress when compared with the sensitive genotype in root length, fresh weight, and dry weight (Fig. 2A-C). In both stress and control conditions, seedling length, fresh weight, and dry weight were highest in the tolerant genotype and had a smaller decrease than the sensitive genotype, which had the greatest decrease. These traits decreased as drought stress intensified, with the tolerant genotype exhibiting a lower percentage decrease (Fig. 2D-F).

### Physiological traits

Analysis of variance (ANOVA) showed a significant difference between stress levels, evaluated genotypes, and the interaction between stress and genotype for the traits chlorophyll a, chlorophyll b, total chlorophyll, and carotenoids (Table 4).

**Table 4:** Variance analysis of physiological traits for sensitive and tolerant genotypes at different levels of drought stress

Sources of variation	Degrees of freedom	Mean Square			
		Chlorophyll a	Chlorophyll b	Carotenoids	Total chlorophyll
Genotype (G)	1	26.859**	8.086**	0.268**	62.258**
Stress treatment (S)	2	28.2**	8.583**	0.689**	67.988**
G × S	2	1.08**	1.023**	0.017**	4.091**
Error	12	0.155	0.034	0.002	0.499
CV%		9.58%	7.32%	7.04%	10.71%

* and ** indicate significance at the 5% and 1% probability levels, respectively.

**Fig. 3:**
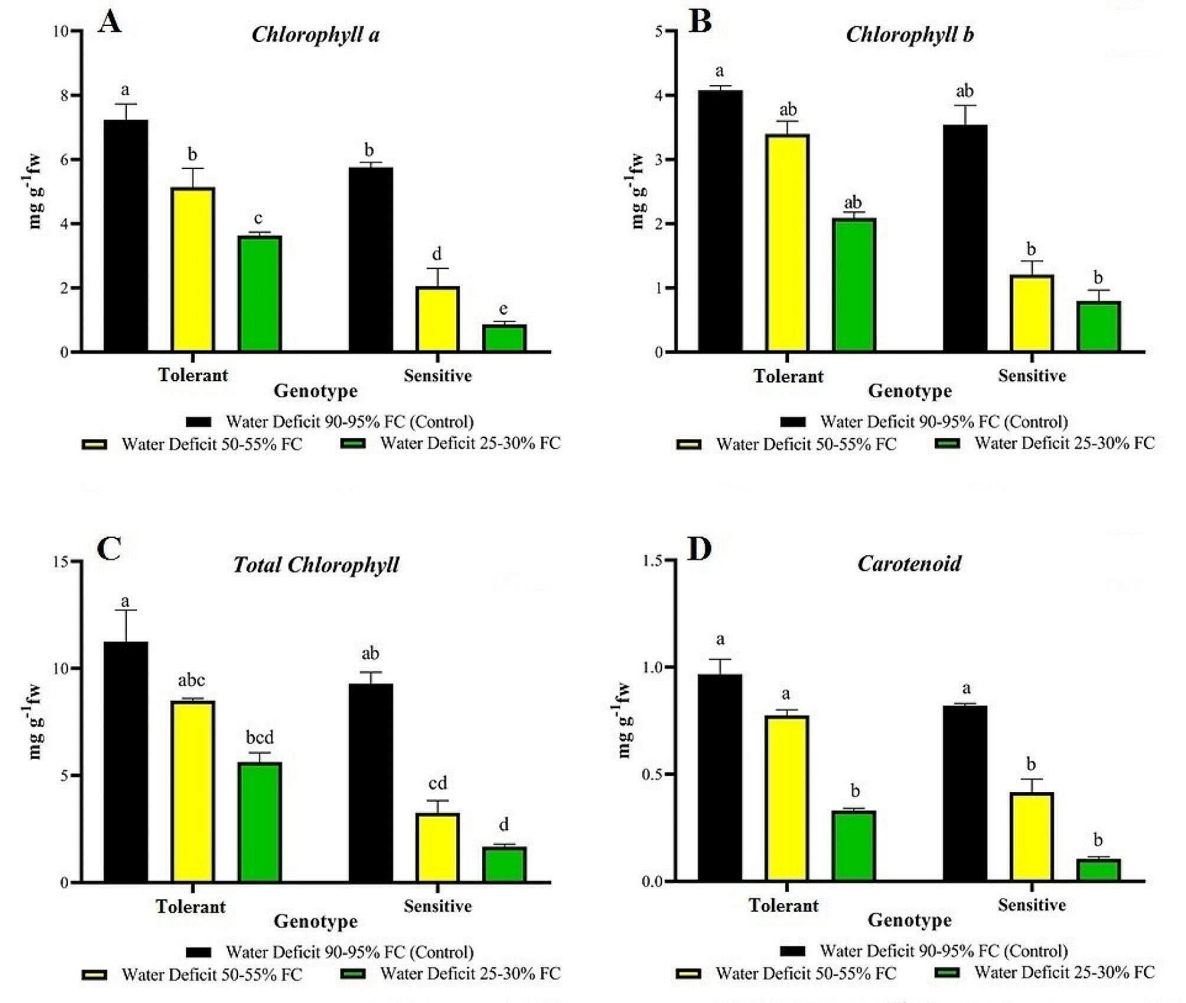
Comparison of physiological traits in sensitive and tolerant genotypes under drought and control conditions

Mean comparison of traits showed that drought stress reduced chlorophyll a, chlorophyll b, total chlorophyll, and carotenoids. This decrease was higher in the susceptible genotype than in the tolerant genotype. The greatest amount of chlorophyll a was found in the tolerant genotype under no stress conditions. In contrast, the susceptible genotype exhibited a significant reduction in chlorophyll a under drought stress, placing it in group e (Fig. 3A).

The amount of chlorophyll b decreased in the sensitive genotype at 25–30% FC and 50–55% FC (Fig. 3B). Total chlorophyll content exhibited the most changes at 25–30% FC. However, these changes were more apparent in the sensitive genotype than in the tolerant genotype. At these levels, the sensitive genotype had the lowest total chlorophyll amount, while the resistant genotype had the highest amount under control conditions (Fig. 3C).

The greatest number of carotenoids was observed in the tolerant genotype under control conditions and at 50–55% FC and in the sensitive genotype under control conditions. The lowest number of carotenoids was observed in the sensitive genotype at 25–30% FC (Fig. 3D).

### Biochemical traits

ANOVA revealed a significant difference between stress levels, evaluated genotypes, and the interaction between stress and genotype in the activity of enzymes CAT, SOD, and POD, and soluble protein (Table 5).

**Table 5:** Analysis of variance of biochemical traits for sensitive and resistant genotypes at different levels of drought stress

Sources of variation	Degrees of freedom	Mean Square			
		POD	SOD	CAT	Protein
Genotype (G)	1	1.919**	26.976**	2.718**	0.328**
Stress treatment (S)	2	7.59**	15.045**	8.019**	0.362**
G × S	2	0.959**	2.121**	1.614**	0.017**
Error	12	0.023	0.227	0.023	0.002
CV%		9.52%	12.61%	10.13%	9.28%

* and ** indicate significance at the 5% and 1% probability levels, respectively.

**Fig. 4:**
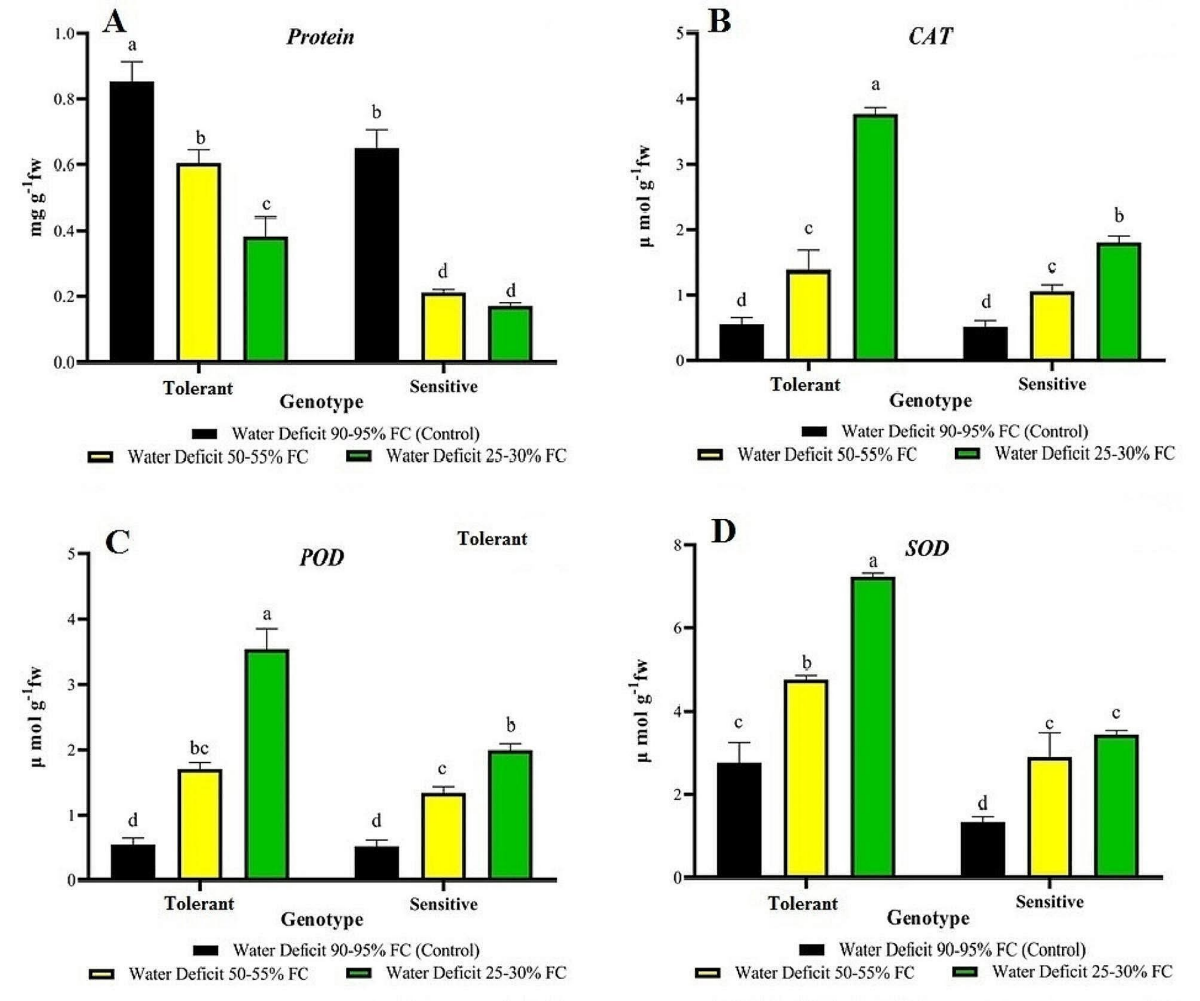
Comparison of biochemical traits in sensitive and tolerant genotypes under drought and control conditions

The amount of soluble protein decreased under drought conditions. Drought stress led to a significant decrease in total protein compared with control conditions. However, this decrease was less pronounced in the tolerant genotype than in the sensitive genotype. The tolerant genotype exhibited the highest amount of protein under control conditions, while the sensitive genotype had the lowest amount at 25–30% FC (Fig. 4A).

The activity of CAT ranged between 3.746 and 0.502 µmol in both drought and control conditions. Drought stress significantly increased CAT activity compared with control conditions. Analysis of variance based on the studied genotypes also revealed a significant difference in CAT levels between genotypes. To further investigate the impact of drought stress on CAT in different genotypes and levels of drought stress, the amount of enzyme was calculated under both stress and control conditions. The tolerant genotype exhibited the highest activity of this enzyme under drought conditions (25–30% FC) (Fig. 4B).

Changes in POD activity was greater under drought conditions than in control conditions, ranging between 3.55 and 0.513 µmol. Overall, drought stress increased POD activity when compared with to control conditions. The tolerant genotype at the 25–30% FC level of stress and the sensitive genotype at 25–30% FC had the greatest effect on POD activity (Fig. 4C).

Drought stress also increased SOD activity compared with control conditions. The greatest SOD activity was observed in the tolerant genotype at 25–30% FC (Fig. 4D).

### Catabolic traits

The ANOVA was conducted to assess stomatal density traits on the surface and underside of the leaf and the stomata length and width on both surfaces. The results revealed a significant difference in these traits among different levels of drought stress, evaluated genotypes, and the interaction between drought stress and genotypes (Table 6). Notably, there was a significant (level of 5%) difference in genotypes for stomatal density traits on both the upper and lower leaf surfaces and in the interaction effect of stress and genotype. Additionally, the interaction effect of genotype in drought stress was also significant at the 5% level for stomatal length and width traits.

**Table 6:** Analysis of variance of catabolic traits for sensitive and tolerant genotypes at different levels of drought stress

Sources of variation	Degrees of freedom	Mean Square					
		Stoma number upper leaves	Stoma number lower leaves	Stoma length upper leaves	Stoma width upper leaves	Stoma length lower leaves	Stoma width lower leaves
Genotype (G)	1	60.5*	24.5*	222.694**	91.312**	189.566**	70.824**
Stress treatment (S)	2	108.5**	39.5**	469.5**	235.927**	437.581**	157.3**
G × S	2	18.5*	24.5*	55.379**	17.061**	39.516*	9.125*
Error	12	4	5	7.111	2.324	6.48	1.662
CV%		7.64%	9.65%	7.27%	9.27%	7.88%	9.52%

* and ** indicate significance at the 5% and 1% probability levels, respectively.

**Fig. 5:**
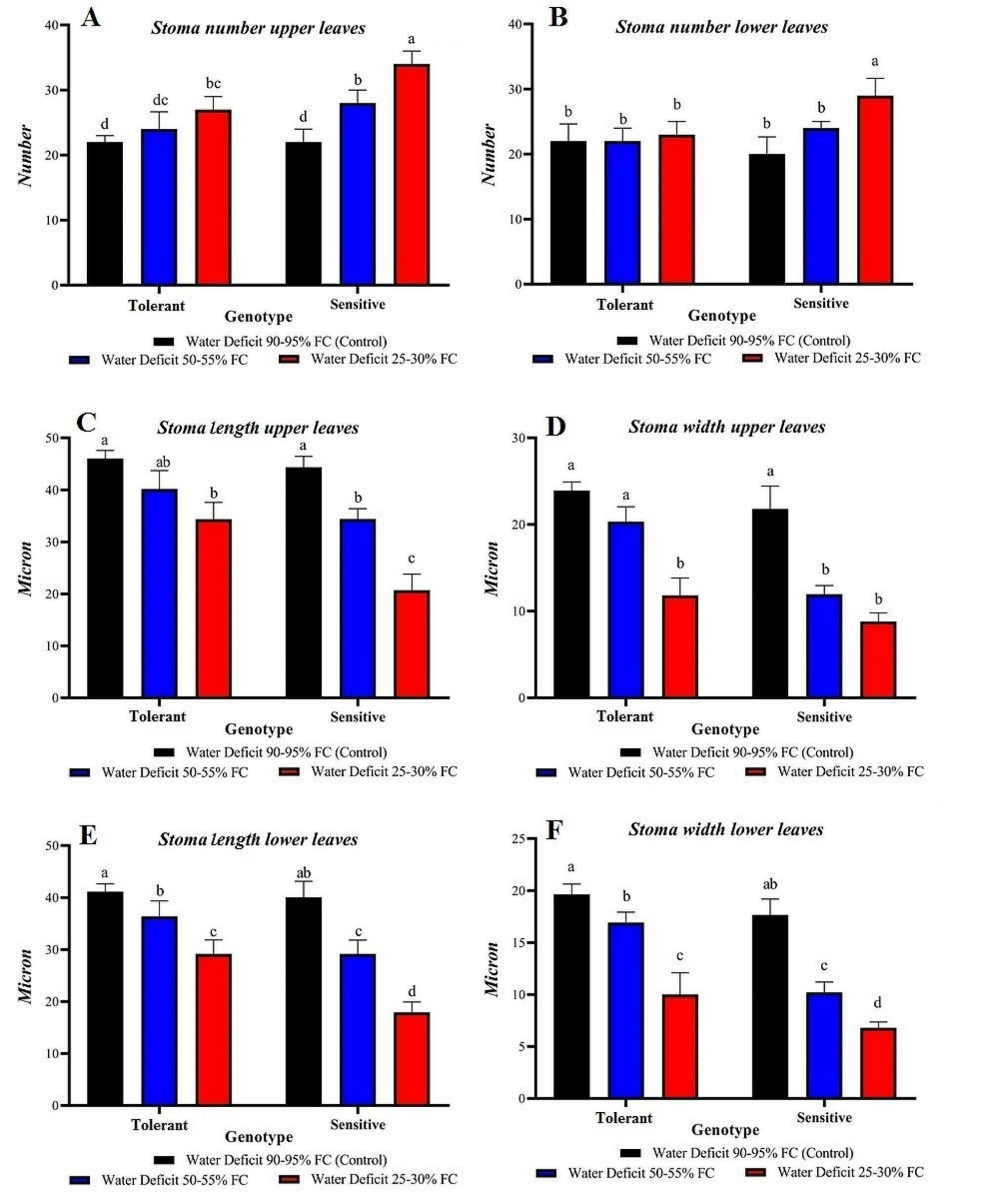
Comparison of catabolic traits in sensitive and tolerant genotypes under drought and control conditions

The sensitive genotype exhibited the highest number of stomata on both upper and lower leaves under control conditions. Drought stress resulted in an increase in stomata number for both the susceptible and tolerant genotypes. However, under drought conditions, the sensitive genotype had a higher number of stomata on both leaf surfaces compared with the tolerant genotype (Fig. 5A-B). The tolerant genotype had greater stomata length and width than the sensitive genotype on both leaf surfaces. These traits decreased as drought stress intensified, but the decrease was less pronounced in the tolerant genotype (Fig. 5C-F).

## Discussion

Among the various environmental stresses, drought is one of the most significant factors that limit growth and production of agricultural plants. Consequently, developing drought-tolerant varieties is crucial for improving yield under such conditions. Screening for drought resistance in extensive genetic collections should be rapid, cost-effective, and non-destructive [20]. Drought tolerance is a quantitative and complex trait that encompasses various morphological, physiological, and biochemical aspects and is controlled by many genes [21]. Therefore, simultaneous selection for all important criteria, considering heritable capabilities and their correlation with drought tolerance, is the most effective method for selecting superior genotypes. In this method, an index is defined with the assistance of all evaluated traits and ideal genotypes are selected based on this single index [22].

The presence of different cycle thresholds (CT) for genes in different drought treatments revealed variations in the expression levels of the studied genes. When examining the melting curves, we observed that each gene in the PCR showed a single peak above the threshold, indicating specific amplification. It is important to note that in some PCRs, a smaller peak was also observed alongside the main peak, which is related to the specific gene amplification. However, we determined that the presence of this smaller peak did not introduce any bias in estimating the concentration of the target gene [23].

Drought stress can induce expression of genes involved in stress tolerance. These genes encode proteins that help the plant cope with drought stress, such as antioxidants, osmolytes, and stress-responsive transcription factors [24]. In the present study, variance analysis of the relative expression of the dihydrine gene and antioxidant gene for each drought condition revealed significant differences between the levels of drought and genotypes. By comparing the averages of the genotypes, we observed that the expression levels of all the studied genes were higher in both drought conditions than in the control conditions. However, the tolerant genotype exhibited the highest level of gene expression under drought conditions compared with the sensitive genotype. Changes in expression, accumulation, and protein synthesis in response to environmental stresses are considered important mechanisms in plants to protect cell metabolism and adapt to their surroundings [25].

Dihydrine can function as an antioxidant and eliminate radicals that are generated within cells during periods of stress. As a result, these proteins may enhance the plant’s ability to withstand stressful conditions. This phenomenon has been observed in transgenic plants that were engineered to carry dihydrine genes. However, it is important to note that while these plants could neutralize hydroxyl and peroxyl radicals, they were unable to eliminate superoxide radicals and hydrogen peroxide [26, 27]. Suprunova et al. [28] reported that drought resistance in wild barley is attributed to expression of various drought-related *Dhn* genes. Among these, *Dhn1* exhibited a more rapid response, while *Dhn6* exhibited a slower reaction to dehydration. Plants produce various types of oxygen-free radicals under control conditions to maintain and establish cellular homeostasis. However, the levels of these radicals significantly increase in stress conditions [29].

To prevent accumulation of these compounds and simultaneously reduce plant growth, it is necessary to activate regulatory mechanisms and scavenging enzymes. Studies on oxidative stress and the activity of antioxidant enzymes in response to drought and osmotic stress indicate that the activity and expression of genes involved in these pathways largely depend on genotype, growth stage, metabolic processes, and stress intensity [30]. In a study conducted on barley plants, expression and activity of SOD and CAT enzymes were higher during the seedling stage compared with the full plant stage, even when no drought stress was present [31]. Drought-tolerant plants may exhibit a range of advantageous characteristics. These include increased dry biomass, higher yields, and greater water potential in their leaves. Additionally, these plants may have higher expression of dihydrine genes when compared with sensitive plants [32]. Therefore, it seems that these proteins are only expressed in plants under drought stress rather than under control conditions [33], or that their accumulation is caused by water deficiency in plants [34]. Therefore, the association of dihydrine with drought tolerance may indicate the presence of a useful protein in the selection of tolerant cultivars [35]. In the present study, we observed an increasing pattern (12.81-fold compared to the control conditions) for *Dhn1* gene under severe drought stress conditions (25–30% FC).

Kaur et al. [36] examined four *Dhn* genes in two genotypes, one tolerant and one sensitive to drought, under drought conditions. They reported a significant increase in expression of the *Wdhn13* gene in tolerant cultivars compared with sensitive cultivars. Additionally, they stated that *Wdhn13* is an abscisic acid (ABA) gene, which is expressed in stress-tolerant cultivars under stress conditions. It appears that this ABA gene is involved in drought tolerance. In addition, Kobayashi et al. [37] and Kurahashi et al. [38] revealed a direct relationship between increased expression of *Dhn* family genes and tolerance to drought and cold stress by ABA.

The root is one of the most crucial plant organs and plays a significant role in acquiring water and nutrients and in aerial organs on the soil surface. The flexibility of the root’s vegetative structure and its ability to develop in response to changes in environmental humidity and soil nutritional status offer an opportunity to assess and explore the natural diversity in germplasm resources. This exploration can help improve plant growth and increase production [39]. Drought stress can damage root cells and reduce the plant’s ability to uptake water and nutrients. Plants can also develop drought-induced root traits, such as deeper rooting and increased root hairs, in response to drought stress [40].

Biomass traits of aerial organs are among the most important characteristics to consider when screening for drought resistance in seedlings. This study revealed a significant level of diversity in the response to drought stress among both sensitive and tolerant wild barley genotypes. Due to the significant differences in these traits, it can be concluded that the studied genotypes have suitable genetic diversity. The significance of the genotype effect indicates the existence of different genetic potential among the studied genotypes for the studied traits. The significant effect of drought stress indicates the impact of different environmental conditions on traits, and the significant interaction effects indicate different reaction trends of genotypes in different environmental conditions. The use of greenhouse conditions and screening a large number of genotypes or cultivars at the seedling stage can lead to identification of useful physiological and biochemical traits related to drought tolerance. In this regard, the results of some studies suggest that observing physiological diversity in the seedling stage may be important in screening and identifying sources of resistance in the full plant stage [41].

Physiological indicators of drought tolerance include the durability of photosynthesis and the maintenance of chlorophyll concentrations under drought conditions. These indicators play a crucial role in stabilizing photosynthesis [42]. Drought stress causes a decrease in the chlorophyll concentration of leaves [43]. According to Mohammadi et al. [44], chlorophyll protein and lipid complexes are less stable in drought-sensitive plants. Drought reduces formation of new plastids and production of chlorophyll a and chlorophyll b, leading to a change in the ratio of chlorophyll a to chlorophyll b. Carotenoids play a crucial role in resistance against environmental stresses. They eliminate oxygen-free radicals, function as non-enzymatic antioxidants, and exhibit a strong correlation with the amount of chlorophyll present in plants [16]. This study also revealed a reduction in chlorophyll and carotenoid content under drought conditions compared with control conditions. Chlorophyll is one of the most crucial photosynthetic components and is highly sensitive to stress conditions [45]. The decreasing trend in chlorophyll content among the evaluated genotypes indicates a high level of genetic variability for this trait. In the present study, drought stress had the greatest impact on the amount of chlorophyll b.

The accumulation of reactive oxygen species produced during stress can damage various cellular compounds, such as DNA, lipids, proteins, chlorophyll, and most significantly, the cell membrane. Ultimately, this accumulation can lead to cell death [46–49]. Drought stress can lead to increased oxidative stress, which is the imbalance between production of reactive oxygen species (ROS) and the ability of the plant to detoxify them. ROSs can damage plant cells and tissues and can also contribute to plant death [50]. Antioxidants protect cells from damage caused by free radicals. Drought stress can increase the production of free radicals in plants. Plants can increase their antioxidant defenses in response to drought stress [51]. Increasing antioxidant enzyme activity in adverse environmental conditions may prevent oxidative stress in cells and degradation of hydrogen peroxide produced in cells. This, in turn, reduces damage to vital biomolecules and helps prevent metabolic disorders [52]. The significance of the antioxidant system in surviving severe dehydration stages is further supported by the commonly observed presence of a robust antioxidant system in regenerative plants [53].

This study showed that stomata length and width on both the upper and lower leaf surfaces decreased in response to drought stress. This decrease was less prominent in the tolerant genotype. By reducing the size of stomatal cells, access to carbon dioxide in plant leaves decreases. This inhibition of photosynthesis subsequently affects plant growth in response to drought [54]. Drought causes the stomata to close, decreasing the rate of photosynthesis and plant growth. This closure leads to a reduction in carbon dioxide concentration in the mesophyll tissue of the leaf, resulting in increased NADPH accumulation [55, 56]. Stomata are specialized epidermal structures that regulate the exchange of water and carbon dioxide between the plant and its surroundings [57]. Maximum efficiency of photosynthesis with minimal water loss requires regulation of the number and position of stomata and the ability to open and close them [58].

Stomata abundance and size of have been extensively studied in selection of drought-tolerant cultivars [59]. The role of stomatal length and width in the rate of water transpiration has been characterized, and differences in stomatal dimensions and number between the lower and upper stomata of leaves have been observed in many plants, including cereals [60]. In certain plants, particularly fodder grasses and cereals, leaf curling serves as a fundamental mechanism for plant resistance against drought stress. However, if this mechanism occurs in leaves with a higher surface concentration of stomata, the amount of photosynthesis and subsequent yield will decrease due to limited gas exchange [61]. This study showed that the number of stomata per unit area increased under drought conditions in both genotypes, with a greater increase observed in the sensitive genotype. It appears that reduction of leaf surface explains the increase in stomata number during drought conditions. Additionally, the tolerant genotype exhibited lower stomatal density in drought conditions compared with the sensitive genotype.

Miskin et al. [62] reported that reducing stomatal density can enhance drought resistance in barley cultivars. Reducing stomata number in response to stress decreases plant access to carbon dioxide, which subsequently lowers its photosynthetic rate [63]. Drought causes osmotic stress by decreasing the water content of leaf cells. In response to water deficit, reduction of stomatal density lowers leaf water loss, thereby preventing the detrimental effects of drought stress [58, 64].

## Conclusions

In this study, we evaluated expression of the *Dhn1*, *SOD*, *POD*, and *CAT* genes in *H. vulgare subsp. spontaneum*. Based on the results of mean comparison, the relative expression levels of *Dhn1*, *SOD*, *POD*, and *CAT* increased under drought stress conditions compared to control conditions. Additionally, the tolerant genotype showed the highest level of expression at 25–30% FC. Furthermore, when comparing the tolerant genotype with both drought stress and non-drought stress conditions, we observed higher amount and a smaller decrease in seedling length, fresh weight, and dry weight; root length, fresh weight, and dry weight; chlorophyll a, chlorophyll b, and total chlorophyll; carotenoids; and soluble protein amount. On the other hand, the sensitive genotype exhibited the greatest decrease in these traits. We conducted tests under both drought stress and non-drought stress conditions to measure the activity levels of SOD, POD, and CAT enzymes. The tolerant genotype exhibited the highest enzyme activity under drought stress conditions (25–30% FC).

## Materials and methods

To select drought-sensitive genotypes, a total of 114 genotypes of wild barley were collected from four western provinces of Iran (Dr Ali Ashraf Mehrabi performed the formal identification) during 2020–2021 and 2021–2022 cropping years under control and stress conditions. More geographical information for the plant material can be found in Supplementary Table S1. The current experiment was conducted using an augmentation design with five replications and nine duplicate parents at the Mahidasht Research Station of the Center for Research and Education of Agriculture and Natural Resources of Kermanshah Province. Based on yield-based indicators [65], drought-tolerant genotype 88-Kozran (Kermanshah, 34.4965° N, 46.5982° E, MSL: 1368, Accession code: IUGB-01657) and sensitive genotype 72-Muchesh (Kurdistan: 35.0571° N, 47.1522° E, MSL: 1368, Accession code: IUGB-01975) were selected using the stress-tolerance scoring index (STS). Selection by the STS index is not based on just one resistance index but on the simultaneous consideration of multiple indices [66, 67].

$${\rm{STS}}\,{\rm{ = }}\,{\rm{GMP}}\,{\rm{ + }}\,{\rm{STI}}\,{\rm{ + }}\,{\rm{HAM}}\,{\rm{ + }}\,{\rm{MP}}\,{\rm{ - }}\,{\rm{TOL}}\,{\rm{ - }}\,{\rm{SSI}}\,{\rm{ - }}\,{\rm{b}}$$

Where b is the linear regression coefficient between the average performance in all environments.

After selection, seeds of each genotype under study were planted in 40 × 20 cm plastic pots in the greenhouse of the Research and Education Center for Agriculture and Natural Resources in Kermanshah province. The growth conditions of the greenhouse were optimized with a light:dark photoperiod of 8:16 and temperature range of 20–25 °C. Each pot contained a 3:1 mixture of sand and agricultural soil. The plant materials were then arranged in a factorial experiment using a completely randomized design. There were two biological replicates for gene expression analysis and three replicates for greenhouse studies.

We considered three levels of drought stress as the first factor and the investigated genotypes as the second factor. We watered the plants regularly, typically two to three times a week, and adjusted watering frequency to plant needs. Once the seedlings had grown and established, we applied drought stress based on the agricultural capacity of the field (FC) at the following three levels: without drought stress (90–95% FC), mild drought stress (50–55% FC), and severe drought stress (25–30% FC). We maintained these conditions until the shoot stage, which is a sensitive stage for barley in terms of drought stress [68].

Drought stress was applied until the time of stem development. Once drought-stress symptoms appeared, necessary preparations were made for sampling and evaluating traits. Sampling was performed during a specific and consistent period for all seedlings. Evaluations were conducted for morphological, biochemical, physiological, and catabolic traits and for gene expression in the leaves under both control conditions and drought stress.

### Preparation of extraction buffer and enzyme extract

To produce enzyme extract for enzyme measurements, 0.1 g of fresh plant tissue was powdered using liquid nitrogen and then poured into 2-ml marked tubes. Next, 1 ml of extraction buffer (prepared the previous day) was added to the plant tissue. These tubes were then stored at 4 °C. The samples were vortexed for 20 s and placed back at 4 °C for 2 h to allow the extraction buffer to perform its activity. The samples were then centrifuged at 4 °C for 20 min at 15 000 g. The supernatant was carefully transferred to 0.2-ml tubes and promptly stored at − 20 °C.

The extract was used to determine the activity of CAT, SOD, and POD. CAT was measured according to Cakmak and Horst [69], POD according to Chance and Maehly [70], and SOD according to Beauchamp and Fridovich [71] using an ELISA device (Bio Tek Power wave). The Bradford method [72] was used to determine protein concentration.

### Chlorophyll and carotenoid contents

The Lichtenthaler and Welburn [73] method was used to measure chlorophyll and carotenoid content. 25 mg of leaves were powdered using a Chinese mortar and liquid nitrogen and then completely homogenized with 2 ml of 96% ethanol in the dark. To fully homogenize the solution, the tubes were shaken and centrifuged at 4 °C for 10 min at 10 000 rpm. The resulting supernatants were poured into a plate and read using an ELISA device (Bio Tek Power wave) at wavelengths 663, 646, and 470 nm. The amount of chlorophyll a, chlorophyll b, total chlorophyll, and carotenoids was calculated using the following formulas:

$${\rm{Chl}}{\mkern 1mu} {\rm{a}}\,{\rm{ = }}\,{\rm{12}}.{\rm{21}}\,\left( {{{\rm{A}}_{{\rm{663}}}}} \right)\,{\rm{ - }}\,{\rm{2}}.{\rm{81}}\,\left( {{{\rm{A}}_{{\rm{646}}}}} \right)$$

$${\rm{Chl}}{\mkern 1mu} {\rm{a}}\,{\rm{ = }}\,20.{\rm{13}}\,\left( {{{\rm{A}}_{{\rm{646}}}}} \right)\,{\rm{ - }}\,5.1\,\left( {{{\rm{A}}_{{\rm{663}}}}} \right)$$

$${\rm{Chl}}\,{\rm{T}}\,{\rm{ = }}\,{\rm{Chl}}\,{\rm{a}}\,{\rm{ + }}\,{\rm{Chl}}\,{\rm{b}}$$

$${\rm{Car}}\,{\rm{ = }}\,\left( {{\rm{1000}}\,{{\rm{A}}_{{\rm{470}}}}\,{\rm{ - }}\,{\rm{3}}{\rm{.27}}\,\left[ {{\rm{Chl}}\,{\rm{a}}} \right]\,{\rm{ - }}\,{\rm{104}}\,\left[ {{\rm{Chl}}\,{\rm{b}}} \right]{\rm{/227}}} \right)$$

### Catabolic traits

To measure stomata number, length, and width in upper and lower leaves, one leaf was randomly chosen from each experimental treatment. A colorless varnish was then applied to the middle section of each leaf. Once the varnish was dry, a piece of adhesive tape was placed on the varnished area to capture the imprint of the stomatal openings. The tape was then placed on a slide, and stomatal characteristics were measured within five randomly selected visual circles using a light microscope at 40x magnification [62].

### Gene-expression analysis

*CAT*, *POD*, *SOD*, and *Dhn1* genes were assessed to investigate the molecular response to drought stress in the selected genotypes. All tools required for RNA extraction were sterilized to inhibit RNases and prevent RNA degradation. RNA extraction was performed using an RNA X Plus kit (CinnaGen, a biotechnology company in Iran). A NanoDrop device (model 2000 C, Thermo Fisher Scientific, USA) was used to determine the quantity, quality, and concentration of RNA. The quality of the extracted total RNA was then assessed by 1% agarose gel electrophoresis.

To eliminate potential contamination of genomic DNA from the extracted RNAs, a DNase1 kit (Thermo Fisher Scientific, USA) was used to treat all RNAs. The cDNA synthesis reaction was performed using a Reverse Transcription Kit (CinnaGen, biotechnology company, Iran). The required components were added to the designated tube of the cDNA production kit and incubated at 55 °C for 60 min. The tubes were then incubated at 95 °C for 5 min in a water bath. The tubes were then placed on ice and transferred to − 80 °C. A Real-Q Plus 2X Master Mix Green kit was used to perform real-time PCR. To assess the efficiency of each primer pair, a combination of all treatments and replicates of the synthesized cDNA was prepared. Various dilution factors were considered for each primer pair. Once the optimal concentration of primer and cDNA was determined, real-time PCR was performed using a Bio-Rad device. The sequence of the primers used along with the reference gene is shown in Table 7.

**Table 7:** Sequence characteristics and melting temperature of primers used in gene-expression analysis

Primer name	Sequence	Temperature (°C)	Band size (bp)
*a -Tubulin-F*	TCCATGATGGCCAAGTGTGA	60	72
*a -Tubulin-R*	GACATCCCCACGGTACATGAG		
*DHN1-F*	GACGAGGGATGGCCACAAGAC	63	443
*DNH1-R*	AGTAACGCATGGCTGCGGATG		
*CAT-F*	GTTCGCCGTCAAGTTTTACA	56	72
*CAT-R*	ATGAAGAAGACGGGGAAGTT		
*SOD-F*	GGGCACCTGAAGATGAAATC	56	120
*SOD-R*	TTGAATTTGGTCCAGTAAGGG		
*POD-F*	AATCAGACCGTCTCCTGCG	59	483
*POD-R*	GGTGGTGTCGTTGTTGAAC		

The relative expression levels of the examined genes were then calculated using the 2^−∆∆CT^ relationship based on the obtained melting temperature for each primer [74].

### Data analysis

A factorial analysis of variance was conducted using a completely random design with four replicates (including two biological replicates and two technical replicates) to analyze relative gene expression levels. GraphPad Prism 8 software was used to perform a variance analysis on greenhouse surveys with a factorial design and a completely random design with three replicates. This software was also used to perform a comparison of average stress levels and the genotypes under study using Duncan’s method at a significant level of effects.

### Electronic supplementary material

Below is the link to the electronic supplementary material.

Supplementary Material 1

## Data Availability

The datasets used and/or analysed during the current study are available from the corresponding author on reasonable request.
