# Supplementary material for: Investigation of the morphological, physiological, biochemical, and catabolic characteristics and gene expression under drought stress in tolerant and sensitive genotypes of wild barley [Hordeum vulgare subsp. spontaneum (K. Koch) Asch. & Graebn.]
Source: BMC Plant Biol. 2024 Mar 26;24:214. doi: 10.1186/s12870-024-04894-z (PMC10964521; doi:10.1186/s12870-024-04894-z)
Supplement: Supplementary file 1 — Supplementary Material 1 [file 12870_2024_4894_MOESM1_ESM.docx]

**Table S1**

Passport of the investigated wild barley in this study

| Genotype number | Accession number | Location/ Region | Geographic coordinate |
| --- | --- | --- | --- |
| 1 | IUGB-00022 | Kermanshah | 46.80317402° E |
|  |  | (Mahidasht) | 34.26700569° N |
| 2 | IUGB-01780 | Kermanshah | 47.02286839° E |
|  |  | (Kermanshah) | 34.29115471° N |
| 3 | IUGB-00795 | Ilam | 47.32094765° E |
|  |  | (Dehloran) | 32.71017776° N |
| 4 | IUGB-00798 | Ilam | 47.37446308° E |
|  |  | (Musian) | 32.50932745° N |
| 5 | IUGB-01783 | Kermanshah | 47.08912969° E |
|  |  | (Halashi) | 34.11056094° N |
| 6 | IUGB-00809 | Ilam | 46.90889597° E |
|  |  | (Shirvan) | 33.70299231° N |
| 7 | IUGB-00984 | Ilam | 47.12868690° E |
|  |  | (Darreh Shahr) | 33.20565867° N |
| 8 | IUGB-01803 | Ilam | 47.44454384° E |
|  |  | (Abdanan) | 32.94097955° N |
| 9 | IUGB-01777 | Ilam | 47.31820107° E |
|  |  | (Darreh Shahr) | 33.11368670° N |
| 10 | IUGB-00850 | Ilam | 46.46534443° E |
|  |  | (Ilam) | 33.65077961° N |
| 11 | IUGB-00873 | Ilam | 47.07924843° E |
|  |  | (Darreh Shahr) | 33.07687091° N |
| 12 | IUGB-00972 | Ilam | 47.10667133° E |
|  |  | (Dehloran) | 32.69096539° N |
| 13 | IUGB-01789 | Kurdistan | 47.01051950° E |
|  |  | (Zarrineh) | 35.99557706° N |
| 14 | IUGB-01805 | Kurdistan | 46.31459355° E |
|  |  | (Saqez) | 36.19957712° N |
| 15 | IUGB-01790 | Kurdistan | 46.23737812° E |
|  |  | (Ziwiye) | 36.08087556° N |
| 16 | IUGB-01751 | Ilam | 47.22481728° E |
|  |  | (Abdanan) | 32.99167538° N |
| 17 | IUGB-01817 | Kurdistan | 47.41158485° E |
|  |  | (Deh Kalan) | 35.29186971° N |
| 18 | IUGB-01807 | Ilam | 47.12381601° E |
|  |  | (Holeylan) | 33.91295029° N |
| 19 | IUGB-01830 | Kurdistan | 47.60109901° E |
|  |  | (Qorveh) | 35.12805197° N |
| 20 | IUGB-01819 | Kermanshah | 46.50650561° E |
|  |  | (Javanrud) | 34.81290485° N |
| 21 | IUGB-01833 | Kermanshah | 46.17073596° E |
|  |  | (Tazeabad) | 34.74861227° N |
| 22 | IUGB-01507 | Lorestan | 48.31791401° E |
|  |  | (Khorramabad) | 33.58330621° N |
| 23 | IUGB-01842 | Kurdistan | 46.05202675° E |
|  |  | (Marivan) | 35.53362318° N |
| 24 | IUGB-01226 | Kermanshah | 45.80011904° E |
|  |  | (Sarpol-e Zahab) | 34.47608421° N |
| 25 | IUGB-01248 | Lorestan | 48.72578144° E |
|  |  | (Borujerd) | 33.88937000° N |
| 26 | IUGB-01794 | Kermanshah | 46.42002583° E |
|  |  | (Gahvareh) | 34.32259832° N |
| 27 | IUGB-01272 | Kermanshah | 46.86871111° E |
|  |  | (Homeyl) | 33.94473062° N |
| 28 | IUGB-01291 | Lorestan | 48.09681416° E |
|  |  | (Selseleh) | 33.92698139° N |
| 29 | IUGB-01298 | Lorestan | 48.22453022° E |
|  |  | (Khorramabad) | 33.58330621° N |
| 30 | IUGB-01801 | Ilam | 47.05858469° E |
|  |  | (Holeylan) | 33.71670092° N |
| 31 | IUGB-01831 | Kermanshah | 47.97733784° E |
|  |  | (Kangavar) | 34.48349553° N |
| 32 | IUGB-01825 | Kermanshah | 45.98775029° E |
|  |  | (Gilanegharb) | 34.17648273° N |
| 33 | IUGB-00028 | Kermanshah | 47.58728564° E |
|  |  | (Harsin) | 34.26539196° N |
| 34 | IUGB-01802 | Kurdistan | 47.57912636° E |
|  |  | (Bijar) | 35.85705704° N |
| 35 | IUGB-01797 | Kermanshah | 47.53321230° E |
|  |  | (Harsin) | 34.23573641° N |
| 36 | IUGB-01823 | Lorestan | 48.93726826° E |
|  |  | (Dorud) | 33.41267234° N |
| 37 | IUGB-01101 | Ilam | 46.92951679° E |
|  |  | (Malekshahi) | 33.17505996° N |
| 38 | IUGB-01822 | Kurdistan | 45.90809941° E |
|  |  | (Baneh) | 36.00706894° N |
| 39 | IUGB-01315 | Lorestan | 47.44312763° E |
|  |  | (Kuhdasht) | 33.55698871° N |
| 40 | IUGB-01325 | Lorestan | 47.12312937° E |
|  |  | (Rumeshkan) | 33.51313224° N |
| 41 | IUGB-01781 | Lorestan | 48.69556904° E |
|  |  | (Zagheh) | 33.49287779° N |
| 42 | IUGB-01784 | Lorestan | 47.57908344° E |
|  |  | (Kuhdasht) | 33.51005502° N |
| 43 | IUGB-01787 | Kermanshah | 46.12679601° E |
|  |  | (Gilanegharb) | 34.08070875° N |
| 44 | IUGB-01785 | Lorestan | 49.04163837° E |
|  |  | (Dorud) | 33.42528097° N |
| 45 | IUGB-01811 | Lorestan | 49.44126606° E |
|  |  | (Azna) | 33.38744960° N |
| 46 | IUGB-01404 | Lorestan | 48.02681923° E |
|  |  | (Veysian) | 33.44891720° N |
| 47 | IUGB-01407 | Lorestan | 47.71920204° E |
|  |  | (Poldokhtar) | 33.13898861° N |
| 48 | IUGB-01786 | Ilam | 46.54703379° E |
|  |  | (Sarableh) | 33.78007476° N |
| 49 | IUGB-01812 | Kurdistan | 46.80317402° E |
|  |  | (Kellaterzan) | 35.32703053° N |
| 50 | IUGB-01791 | Kermanshah | 46.37063026° E |
|  |  | (Gwawar) | 33.95389753° N |
| 51 | IUGB-01829 | Ilam | 46.19210243° E |
|  |  | (Mehran) | 33.08607630° N |
| 52 | IUGB-01491 | Lorestan | 47.19315648° E |
|  |  | (Darb-e Gonbad) | 33.71423781° N |
| 53 | IUGB-01507 | Lorestan | 48.23002338° E |
|  |  | (Khorramabad) | 33.40006189° N |
| 54 | IUGB-01806 | Kermanshah | 46.22231483° E |
|  |  | (Kerend-e Gharb) | 34.30175539° N |
| 55 | IUGB-01345 | Lorestan | 47.62714863° E |
|  |  | (Kuhdasht) | 33.62219564° N |
| 56 | IUGB-01351 | Kermanshah | 45.87349892° E |
|  |  | (Gilanegharb) | 34.26090528° N |
| 57 | IUGB-01818 | Lorestan | 49.09657001° E |
|  |  | (Dorud) | 33.45049273° N |
| 58 | IUGB-01832 | Kermanshah | 46.32119179° E |
|  |  | (Srmast) | 34.05180606° N |
| 59 | IUGB-01834 | Lorestan | 47.97459126° E |
|  |  | (Dowreh) | 33.58330621° N |
| 60 | IUGB-01838 | Ilam | 46.77464604° E |
|  |  | (Lowmar) | 33.59335194° N |
| 61 | IUGB-00813 | Ilam | 46.98648691° E |
|  |  | (Shirvan) | 33.59953610° N |
| 62 | IUGB-01651 | Ilam | 46.69468403° E |
|  |  | (Malekshahi) | 33.21068561° N |
| 63 | IUGB-01667 | Lorestan | 47.83181190° E |
|  |  | (Poldokhtar) | 33.04464445° N |
| 64 | IUGB-01843 | Ilam | 48.30967426° E |
|  |  | (Chagha Sabz) | 33.32895055° N |
| 65 | IUGB-01800 | Ilam | 46.62327290° E |
|  |  | (Malekshahi) | 33.04968061° N |
| 66 | IUGB-01028 | Ilam | 47.32644081° E |
|  |  | (Abdanan) | 32.82796161° N |
| 67 | IUGB-01804 | Kurdistan | 47.02427387° E |
|  |  | (Sanandaj) | 35.33039172° N |
| 68 | IUGB-00837 | Ilam | 47.48024940° E |
|  |  | (Darreh Shahr) | 33.06996623° N |
| 69 | IUGB-01799 | Kurdistan | 46.96519017° E |
|  |  | (Abidar) | 35.31305909° N |
| 70 | IUGB-01826 | Lorestan | 48.74363422° E |
|  |  | (Zagheh) | 33.45278434° N |
| 71 | IUGB-01385 | Lorestan | 49.40834999° E |
|  |  | (Azna) | 33.49702927° N |
| 72 | IUGB-01975 | Kurdistan | 47.14886248° E |
|  |  | (Muchesh) | 35.05467940° N |
| 73 | IUGB-01820 | Kermanshah | 46.71120644° E |
|  |  | (Homeyl) | 33.89236242° N |
| 74 | IUGB-01798 | Kurdistan | 46.93754733° E |
|  |  | (Kamyaran) | 34.80444816° N |
| 75 | IUGB-01603 | Kermanshah | 46.53542519° E |
|  |  | (Eslamabad-e-Gharb) | 34.13823589° N |
| 76 | IUGB-01821 | Lorestan | 47.60929585° E |
|  |  | (Kuhdasht) | 33.75817041° N |
| 77 | IUGB-01810 | Kurdistan | 46.27270818° E |
|  |  | (Saqez) | 36.27296075° N |
| 78 | IUGB-01177 | Lorestan | 48.54592323° E |
|  |  | (Chaghalvandi) | 33.67092561° N |
| 79 | IUGB-01782 | Kurdistan | 46.26626015° E |
|  |  | (Marivan) | 35.50456163° N |
| 80 | IUGB-01326 | Lorestan | 48.14075947° E |
|  |  | (Khorramabad) | 33.44820105° N |
| 81 | IUGB-01815 | Kurdistan | 45.86381078° E |
|  |  | (Baneh) | 35.98845889° N |
| 82 | IUGB-01835 | Kermanshah | 46.34316444° E |
|  |  | (Paveh) | 35.06962614° N |
| 83 | IUGB-01849 | Kermanshah | 46.68648720° E |
|  |  | (Ravansar) | 34.72045088° N |
| 84 | IUGB-01844 | Kurdistan | 47.14241982° E |
|  |  | (Divandarreh) | 35.92381047° N |
| 85 | IUGB-01836 | Kurdistan | 46.22231483° E |
|  |  | (Bukan) | 36.50660067° N |
| 86 | IUGB-01813 | Kurdistan | 47.11220741° E |
|  |  | (Takab) | 36.40277064° N |
| 87 | IUGB-01792 | Kermanshah | 47.63955116° E |
|  |  | (Sonqor) | 34.73850913° N |
| 88 | IUGB-01657 | Kermanshah | 46.52169228° E |
|  |  | (Kuzaran) | 34.53286394° N |
| 89 | IUGB-01816 | Kurdistan | 46.33767128° E |
|  |  | (Sarvabad) | 35.30531948° N |
| 90 | IUGB-01808 | Kurdistan | 46.22395635° E |
|  |  | (Saqez) | 36.22339973° N |
| 91 | IUGB-01814 | Kurdistan | 46.60126805° E |
|  |  | (Palangan) | 35.06530574° N |
| 92 | IUGB-01837 | Kurdistan | 47.86751747° E |
|  |  | (Qorveh) | 35.19092524° N |
| 93 | IUGB-01809 | Kurdistan | 46.93642616° E |
|  |  | (Divandarreh) | 35.89711585° N |
| 94 | IUGB-01793 | Kurdistan | 46.95970774° E |
|  |  | (Hasan Abad) | 35.25438071° N |
| 95 | IUGB-01795 | Kermanshah | 47.40883827° E |
|  |  | (Harsin) | 34.24501376° N |
| 96 | IUGB-01839 | Ilam | 47.26734638° E |
|  |  | (Dehloran) | 32.60655534° N |
| 97 | IUGB-01796 | Kurdistan | 46.90683603° E |
|  |  | (Kamyaran) | 34.80513530° N |
| 98 | IUGB-01840 | Kermanshah | 45.62355995° E |
|  |  | (Qasreshirin) | 34.49439022° N |
| 99 | IUGB-00883 | Ilam | 46.42414570° E |
|  |  | (Ilam) | 33.59017032° N |
| 100 | IUGB-01845 | Kurdistan | 47.96364784° E |
|  |  | (Chahardoli) | 34.95264896° N |
| 101 | IUGB-00997 | Ilam | 46.85671091° E |
|  |  | (Shirvan) | 33.72469660° N |
| 102 | IUGB-01848 | Kurdistan | 46.31295204° E |
|  |  | (Sarvabad) | 35.31428475° N |
| 103 | IUGB-01841 | Kermanshah | 47.16158152° E |
|  |  | (Kermanshah) | 34.38120015° N |
| 104 | IUGB-01534 | Lorestan | 47.71366596° E |
|  |  | (Kuhdasht) | 33.55355540° N |
| 105 | IUGB-01543 | Kermanshah | 45.65102577° E |
|  |  | (Gilanegharb) | 34.06090810° N |
| 106 | IUGB-01702 | Ilam | 46.78666234° E |
|  |  | (Tulab) | 33.71852143° N |
| 107 | IUGB-01827 | Lorestan | 48.43739033° E |
|  |  | (Khorramabad) | 33.48829662° N |
| 108 | IUGB-01846 | Lorestan | 49.01691914° E |
|  |  | (Dorud) | 33.51692495° N |
| 109 | IUGB-01628 | Ilam | 46.76022649° E |
|  |  | (Lowmar) | 33.53613641° N |
| 110 | IUGB-01079 | Ilam | 46.74272776° E |
|  |  | (Shirvan) | 33.68128253° N |
| 111 | IUGB-01828 | Kermanshah | 47.72194862° E |
|  |  | (Sahneh) | 34.49891746° N |
| 112 | IUGB-01788 | Kermanshah | 47.27150917° E |
|  |  | (Bisotun) | 34.45136916° N |
| 113 | IUGB-01847 | Lorestan | 49.59782124° E |
|  |  | (Aligudarz) | 33.41840376° N |
| 114 | IUGB-01824 | Lorestan | 47.78512001° E |
|  |  | (Poldokhtar) | 32.99167538° N |
